# Experience sampling reveals the role that covert goal states play in task-relevant behavior

**DOI:** 10.1038/s41598-023-48857-0

**Published:** 2023-12-07

**Authors:** Brontë Mckeown, Will H. Strawson, Meichao Zhang, Adam Turnbull, Delali Konu, Theodoros Karapanagiotidis, Hao-Ting Wang, Robert Leech, Ting Xu, Samyogita Hardikar, Boris Bernhardt, Daniel Margulies, Elizabeth Jefferies, Jeffrey Wammes, Jonathan Smallwood

**Affiliations:** 1 Psychology Department, Queen’s University, Kingston, K7L 3N6 Canada; 2 Neuroscience, Brighton and Sussex Medical School, University of Sussex, Brighton, BN1 9RH UK; 3 CAS Key Laboratory of Behavioural Science, Institute of Psychology, Chinese Academy of Sciences, Beijing, 100101 China; 4 Department of Psychiatry and Behavioral Sciences, Stanford University, Stanford, CA 94305 USA; 5 Department of Psychology, Durham University, Durham, DH1 3LE UK; 6 School of Psychology, University of Sussex, Brighton, BN1 9QH UK; 7 Centre de Recherche de l’institut Universitaire de Gériatrie de Montréal (CRIUGM), Montreal, QC H3W 1W5 Canada; 8 Centre for Neuroimaging Science, King’s College, London, SE5 8AF UK; 9 Center for the Developing Brain, Child Mind Institute, New York, 10022 USA; 10 Department of Neurology, Max Planck Institute for Human Cognitive and Brain Sciences, 04103 Leipzig, Germany; 11 Montreal Neurological Institute, McGill University, Montreal, H3A 2B4 Canada; 12 Integrative Neuroscience and Cognition Center (UMR 8002, Centre National de la Recherche Scientifique (CNRS) and Université de Paris, 75006 Paris, France; 13 Department of Psychology, University of York, York, YO10 4LX UK

**Keywords:** Cognitive neuroscience, Human behaviour

## Abstract

Cognitive neuroscience has gained insight into covert states using experience sampling. Traditionally, this approach has focused on off-task states. However, task-relevant states are also maintained via covert processes. Our study examined whether experience sampling can also provide insights into covert goal-relevant states that support task performance. To address this question, we developed a neural state space, using dimensions of brain function variation, that allows neural correlates of overt and covert states to be examined in a common analytic space. We use this to describe brain activity during task performance, its relation to covert states identified via experience sampling, and links between individual variation in overt and covert states and task performance. Our study established deliberate task focus was linked to faster target detection, and brain states underlying this experience—and target detection—were associated with activity patterns emphasizing the fronto-parietal network. In contrast, brain states underlying off-task experiences—and vigilance periods—were linked to activity patterns emphasizing the default mode network. Our study shows experience sampling can not only describe covert states that are unrelated to the task at hand, but can also be used to highlight the role fronto-parietal regions play in the maintenance of covert task-relevant states.

## Introduction

Contemporary cognitive neuroscience has established the neural correlates of hidden states such as mind-wandering^1^, or more recently, mind-blanking^2^, by associating brain activity with patterns of thought described by experience sampling^3–5^. These states are a natural target for experience sampling studies because they help establish the correlates of states that occur spontaneously, and so are hard to evoke using standard experimental methods^6–8^. However, overt task behavior can also depend on the maintenance of task-relevant information in awareness^9^. Accordingly, it is possible that experience sampling can also illuminate the nature of covert processes that are hypothesized to help task performance. Consistent with this possibility, Turnbull et al.^10^ established that when people are off-task, regions involved in external attention, such as regions of the dorsal lateral prefrontal cortex (dLPFC), show reductions in activity, and that in demanding situations, regions of the dLPFC can suppress off-task activity. However, we currently lack an understanding of the patterns of brain activity that support the active maintenance of deliberate task-relevant information covertly in awareness.

To address this gap in our understanding, we re-analyzed a published data set by Konu et al.^11^ to examine whether a pattern of deliberate task-relevant focus is related to specific patterns of neural activity and is linked to better performance on the task at hand. To achieve this goal, we developed a neural state space based on dimensions of brain function variation, derived from applying dimension reduction techniques to resting-state connectivity data and commonly referred to as ‘gradients’^12^. These gradients depict biologically relevant axes that differentiate observed function in major brain systems (for a review, see^13^) and provide an organizing framework for the relationships between large-scale networks^8,12^. We used these gradients to build a 5-d coordinate system that allows us to organize whole-brain maps in a ‘common space’, in which the relative locations within this space provide information regarding the balance of different macroscale systems in a particular context (see also^14–16^). This analytic approach is focused on how different large-scale systems interact together and so provides a biologically relevant macroscale perspective on brain states^17^ that complements perspectives that focus on parcels^18^ and large-scale networks^19^ (see Konu et al.^11^ for a region-based analysis of the data used in the current study).

In the current study, we use this common space to examine how macroscale patterns of brain activity are related to both (1) covert experiential states that emerge during task processing and that we index via experience sampling and (2) the implementation of different stages of goal-relevant behavior that occur during task completion. The task was a simple sustained attention task in which participants were asked to ignore (frequently presented) non-targets and respond to (infrequent) targets. We chose this task situation because prior studies (e.g.,^20^) have established that in tasks with relatively low demands, a balance of both task-focused states and self-generated states can emerge, providing the opportunity to realize both task-relevant and task-irrelevant covert states. To index covert experiential states during the task, multidimensional experience sampling (mDES)^8,21^ was employed, a method that requires individuals to intermittently describe their thoughts by rating several dimensions (e.g., level of task focus). This technique has previously been used to identify covert experiential states, including the deliberate maintenance of task-relevant information, as well as patterns of thought that are less related to the here and now, including thoughts with a social episodic focus, and patterns of thought with different modalities (verbal or visual) (e.g.,^10,11,22,23^). This approach is also sensitive to changes in neural function as indexed by functional magnetic resonance imaging (fMRI)^8,11,14,24–34^.

**Figure 1:**
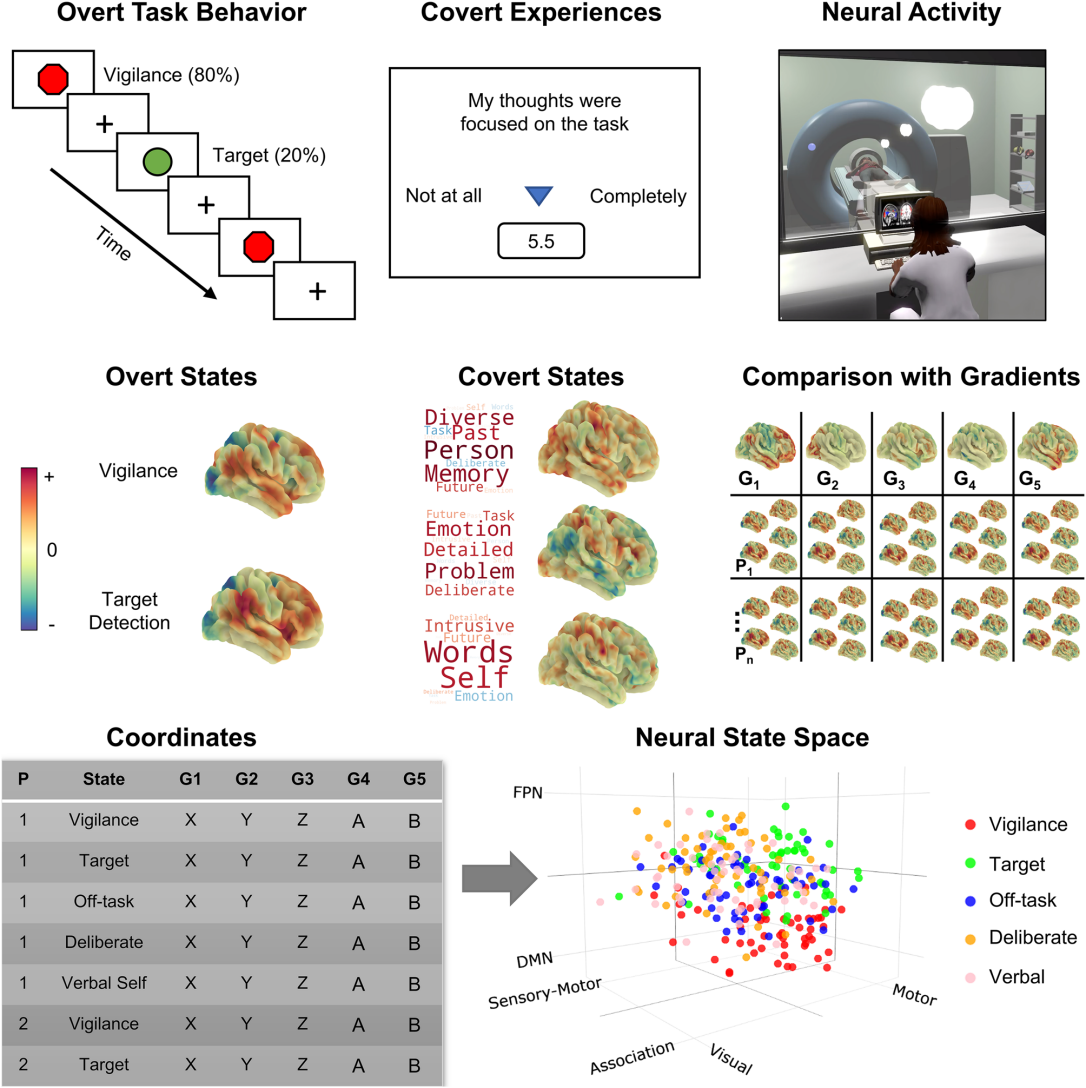
Schematic illustration of the task paradigm and locating the overt task states and covert experiential states in the neural state space. Top row depicts the task paradigm. Participants completed a sustained attention task while brain activity was measured using fMRI. The task comprised of vigilance periods, in which non-target shapes were presented (80% of trials), and periods of target detection, in which target shapes were presented, requiring a behavioral response (20% of trials). During each task run, (13 min × 3), 8 experience sampling probes were presented. Each probe asked participants to rate 13 items regarding their covert experience during the task (e.g., level of task focus; see Supplementary Table 8). Principal components analysis applied to this data identified three thought patterns, represented as word clouds in the second panel (each word = experience sampling item, size = magnitude of component loading for that item, and color = direction; warm = positive, cool = negative). Our experimental approach, therefore, allowed us to identify brain correlates of overt task states (vigilance and target detection) and covert states (assessed via experience sampling). Brain maps for overt and covert states (second row) were calculated via the application of the general linear model to each participant using the task time-course, and the time-course for each thought pattern, as explanatory variables (calculated at the first-level using FSL; see “Materials and methods”). In these maps, warmer colors correspond to positive values, while cooler colors correspond to negative values (each brain map has its own color scale). Having identified covert and overt brain states for each individual, we performed pairwise correlations between each of the five connectivity gradients and each individual’s covert and overt brain maps (right-hand side of second row) to produce, for each person and each map, five gradient coordinates, indicating where each individual’s brain maps fall in the 5-d neural state space (left-hand side of third row). The results of this analysis are represented in the 3-d scatterplot, in which each point represents an individual’s brain maps location in the state space (N observations = 285). Different colored dots describe different types of brain-cognition relationships.

**Figure 2:**
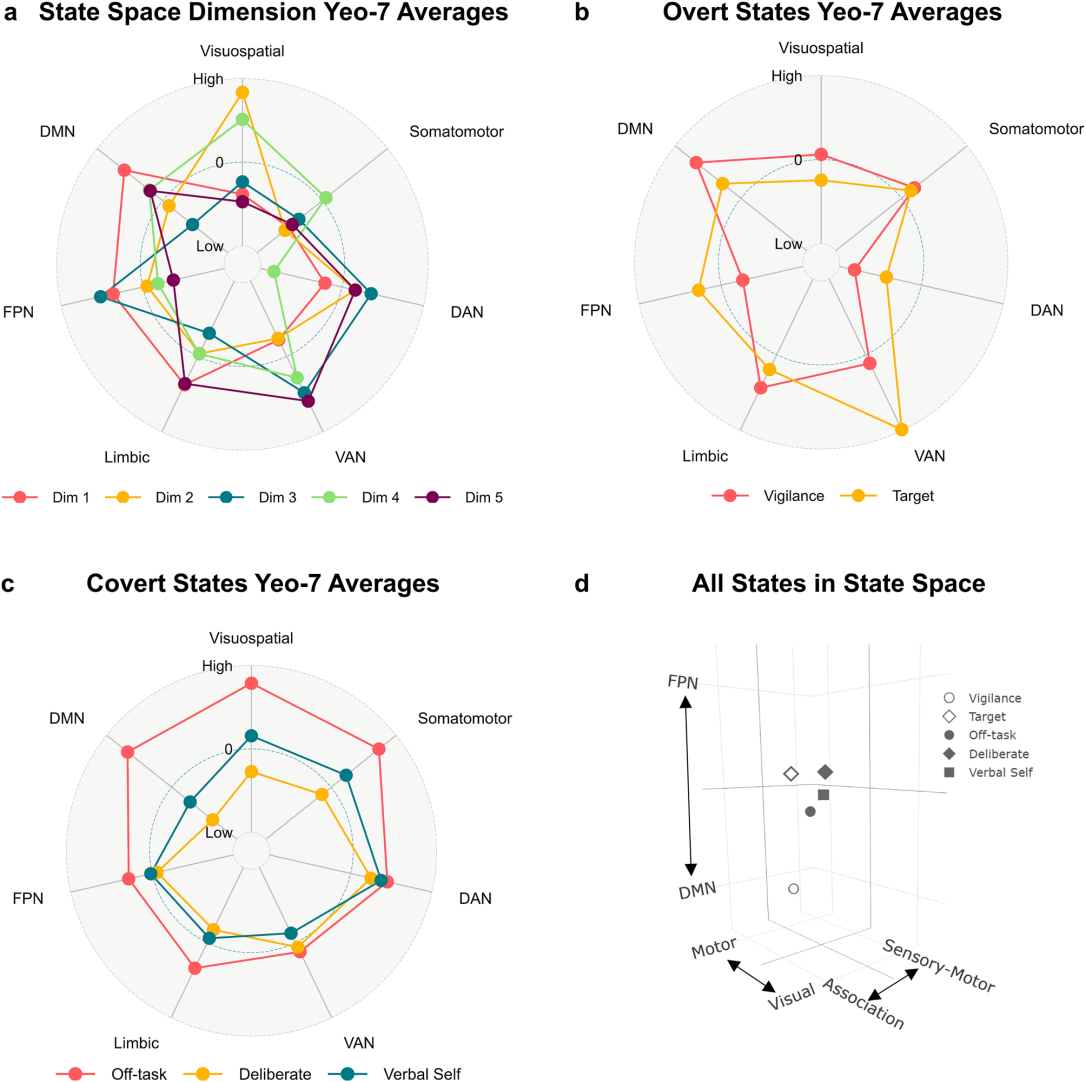
Network configuration of the state space dimensions and the overt and covert brain states, and the group-average location of each brain state along the first three dimensions of the state space. (**a**) A radar plot showing the average gradient value in each of the Yeo-7 brain networks^36^ for each of the five dimensions that make up the 5-d state space. To allow comparison of network configuration along each state space dimension, the network-averaged values were z-scored within each gradient prior to plotting. The maximum value (‘High’) is 3 and the minimum value (‘Low’) is -3. Each color represents a different dimension, as shown in the legend at the bottom of the plot (*Dim* dimension, *DAN* dorsal attention network, *VAN* ventral attention network, *FPN* fronto-parietal network, *DMN* default mode network). (**b**) A radar plot showing the average z-stat value in each of the Yeo-7 brain networks^36^ for each of the group-level overt task brain states (vigilance and target detection). The maximum value (‘High’) is 4.5 and the minimum value (‘Low’) is − 4.5. Each color represents a different group-level brain state, as shown in the legend at the bottom of the plot. (**c**) A radar plot showing the average z-stat value in each of the Yeo-7 brain networks^36^ for each of the group-level covert experiential brain states (off-task social episodic cognition, deliberate task focus, and verbal, self-relevant thought). The maximum value (‘High’) is 1.5 and the minimum value (‘Low’) is − 1.5. Each color represents a different group-level brain state, as shown in the legend at the bottom of the plot. (**d**) A 3-d plot showing the group-average location of each (individual-level) brain state examined in the current study along the first three dimensions of the state space. Open shapes represent overt task states (vigilance and target detection) while closed shapes represent covert states (off-task social episodic cognition, deliberate task focus, and verbal, self-relevant thought). The networks at the extreme ends of each dimension are plotted as axis labels and each axis ranges from − 0.3 to 0.3.

We used these data to understand whether experience sampling can be used to identify the neural correlates of patterns of thought that are linked to the organization of task-relevant behavior. First, to generate our neural state space, we selected the first five connectivity gradients^12^ calculated from resting-state data collected as part of the Human Connectome Project (HCP)^35^, accounting for approximately 60% of the connectivity variance. Our approach, similar to prior published work^14–16^, uses gradients calculated from the HCP data to form the dimensions of the state space—rather than gradients calculated from the specific data set in question—because this prevents the dimensions from emphasizing certain features of our study’s specific population. Moreover, by using gradients from the HCP as a set of canonical gradients to form our state space, it will be possible in the future to be able to directly relate studies focused on different questions, such as tasks or rest (e.g.,^14,15^), and from different research groups^16^, thereby providing an important tool for understanding brain states moving forward. The first dimension differentiates sensory-motor cortex from association cortex, the second dimension describes differences between motor and visual cortex, the third dimension differentiates regions of the default mode network from the fronto-parietal network, the fourth dimension differentiates regions of the dorsal attention network from regions of the ventral attention network and visual cortex, and the fifth dimension differentiates regions of the visual cortex from the ventral attention network. The combination of these five gradients form a 5-d state space (the first three dimensions are presented in Fig. 1, and, the network configuration of all five dimensions is shown in Fig. 2).

We used these gradients to organize the overt and covert features of cognition derived from our data by projecting the relevant task and experiential maps for each participant into the 5-d space, correlating each map with each gradient dimension (see Fig. 2, panels a and b, for radar plots showing the network configuration of the group-level maps for each brain state examined in the current study). This analytic step resulted in a set of five ‘coordinates’ for each task and experiential map for each participant, in which each coordinate describes the location of each map along each dimension (the group-averaged locations for each state along the first three dimensions are shown in Fig. 2, panel d). Finally, we used a series of linear mixed models to apply inferential statistics to these coordinates. This analytical pipeline is shown in Fig. 1 (see “Materials and methods” for further details).

## Results

### Location of overt task states in the neural state space and how these locations relate to target detection performance

**Figure 3:**
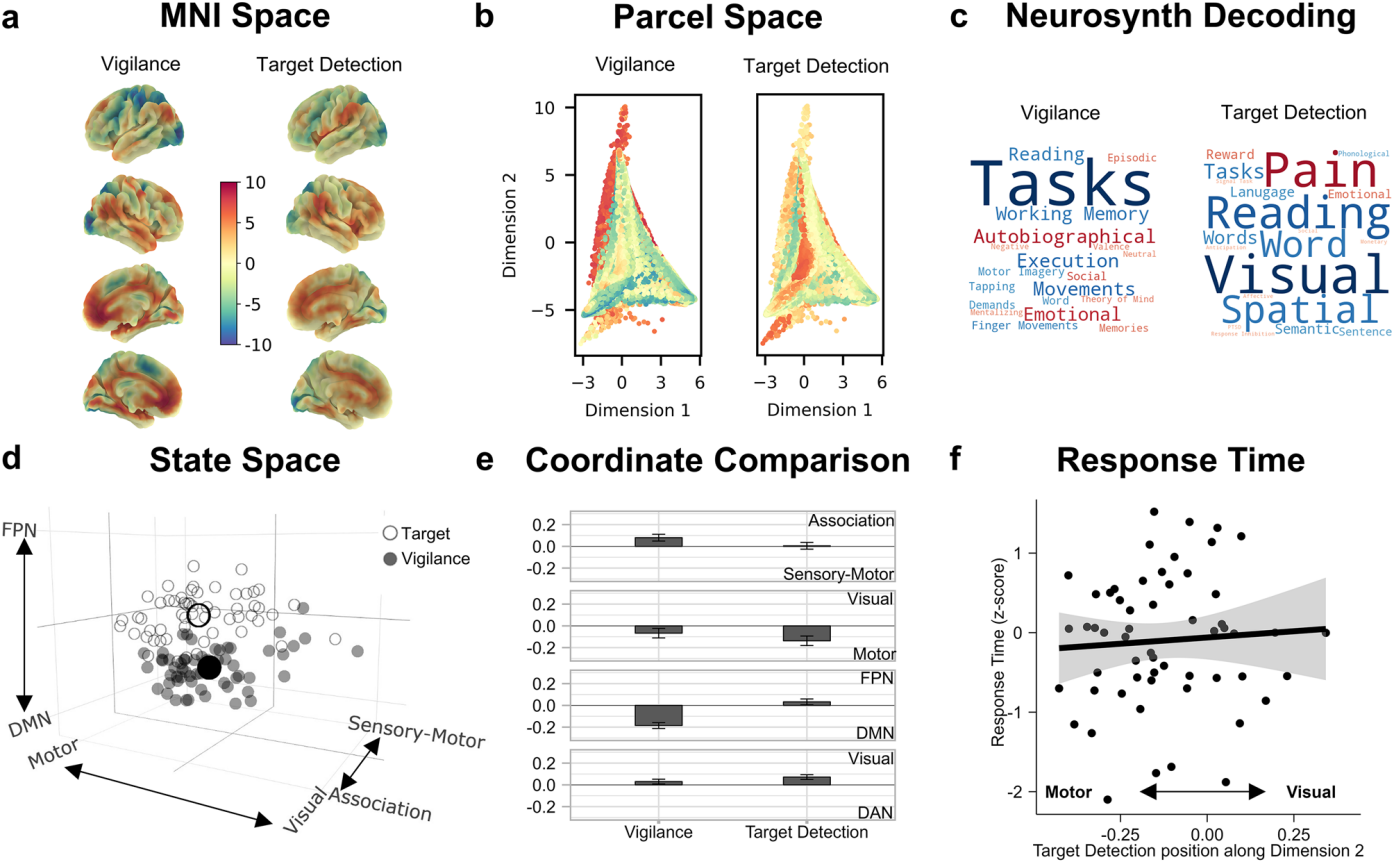
Location of overt task events in the neural state space and their relationship to task performance. (**a**) The (unthresholded) target and vigilance group-level maps plotted on the cortical surface in MNI space (N = 57). These two maps share a common color scale. (**b**) A scatter plot of the first two dimensions of the state space (points = parcels), colored by the fMRI BOLD activity in the target and vigilance brain maps shown in panel (**a**). (**c**) Word clouds representing the results from a meta-analysis using Neurosynth to decode the most likely terms used to describe the pattern of brain activity seen in the target and vigilance maps, where the size of the word represents the magnitude of the relationship, and the color represents the direction (warmer colors = positive relationship, cooler colors = negative relationship). (**d**) 3-d scatterplot showing where (1) individual target and vigilance maps fall in the state space (smaller circles) and (2) the average position of these maps across the sample (larger circles). In this plot, each small point represents a whole-brain map for each task condition for each participant (N observations = 114). Open circles represent target maps while closed circles represent vigilance maps. Axis labels represent the systems at the extreme end of each dimension. Axis values range from − 0.5 to 0.5. (**e**) Bar graph showing the significant results of the linear mixed models comparing the state space coordinates of vigilance and target detection states along each dimension in the neural state space. Each bar represents the estimated marginal mean (i.e., predicted value) for each level of 'task map' (vigilance and target detection). Error bars represent 95% CIs (N observations = 114). Y-axis labels shown on the right-hand-side indicate the brain systems at the extreme ends of each dimension. (**f**) Scatterplot showing the relationship between target detection response time (z-scored seconds; y-axis) and the position of the target detection states along the motor to visual dimension of the state space (dimension 2; x-axis). Error bars represents 95% CIs (N observations = 57). X-axis labels indicate extreme ends (motor and vision) of dimension 2.

Our first goal was to determine how the neural state space differentiated the two features of overt behavior in our task paradigm (vigilance and target detection) and to understand whether their positions in the state space relate to task performance. To this end, we first calculated the pairwise correlations between spatial brain maps summarizing neural activity during periods of vigilance and target detection with each of the five connectivity gradients, resulting in five sets of coordinates per task map for each individual (see “Materials and methods”). The results of this process can be seen in Fig. 3 (panel d).

To understand how these task events are differentiable along the five dimensions of our state space, we compared the position (i.e., the coordinates) of these maps along each dimension in a series of linear mixed models (see Supplementary Fig. 1 for the distribution of these coordinates for each task map along each dimension). We also examined how these positions in the state space related to target detection performance during the task in a multiple regression in which the task maps’ coordinates were the explanatory variables and response time was the outcome variable. In addition to traditional significance testing of main effects, bootstrapping (n iterations = 1000) was used to calculate parameter estimates and their associated confidence intervals and P values to establish the robustness of results emerging from these models (see “Materials and methods”). Reported P values in-text are unadjusted, unless specified in parentheses.

The mixed models comparing the coordinates of each task map along each dimension of the state space indicated that the position of vigilance and target detection states differed significantly along each of the first four dimensions [dimension 1: [*F*(1, 56) = 18.22, *P* < 0.001]; dimension 2: [*F*(1, 56) = 9.89, *P* = 0.003]; dimension 3: [*F*(1, 56) = 198.51, *P* < 0.001]; dimension 4: [*F*(1, 56) = 11.98, *P* = 0.001]].

Along dimension 1—which separates sensory-motor and association cortex—vigilance states, compared to target detection states, were located further towards the association end [bootstrapped *b* = 0.04, 95% CI [0.02, 0.05], *P* < 0.001]. This pattern of results confirms the broad hypothesis that states falling toward the sensory-motor end of the principal gradient are likely to be more important for immediate action^37^. Along dimension 2—which separates motor and visual systems—target detection states, compared to vigilance states, were located further towards the motor end [bootstrapped *b* = − 0.03, 95% CI [− 0.06, − 0.01], *P* < 0.001]. Along dimension 3—which separates the default mode and fronto-parietal networks—vigilance states, compared to target detection states, were located further towards the default mode end [bootstrapped *b* = − 0.11, 95% CI [− 0.12, − 0.09], *P* < 0.001], a pattern that is consistent with prior views of the default mode network as especially important for passive states (often referred to as the sentinel hypothesis^38^). Finally, along dimension 4—which separates the dorsal attention network from regions of visual cortex and ventral attention network—target detection states, compared to vigilance states, were located further towards the visual and ventral attention end [bootstrapped *b* = 0.02, 95% CI [0.01,0.03], *P* < 0.001]. In our paradigm, where behavioral responses are relatively infrequent, the association between the ventral attention network and target detection is consistent with evidence of this system being important for responding to salient events in the environment^39^.

Overall, therefore, vigilance states tended to fall towards the association end of dimension 1, the visual end of dimension 2, the default mode end of dimension 3, and the dorsal attention network end of dimension 4. In contrast, target detection states tended to fall towards the sensory-motor end of dimension 1, the motor end of dimension 2, the fronto-parietal end of dimension 3, and the visual and ventral attention end of dimension 4 (see Fig. 3e). All bootstrapped parameter estimates for these models are presented in Supplementary Table 1.

Finally, the multiple regression indicated that individuals whose target detections states fell further towards the motor end of the motor to visual dimension tended to respond faster to the targets [*F*(1, 43) = 4.69, *P* = 0.040; bootstrapped *b* = − 2.00, 95% CI [− 3.72, − 0.29], *P* = 0.016] (see Fig. 3f for scatterplot) and, while a less consistent relationship, in which the bootstrapped estimates overlapped with zero, individuals whose vigilance states fell further towards the association end of the sensory-motor to association dimension tended to respond faster to targets [*F*(1, 43) = 4.62, *P* = 0.040; bootstrapped *b* = − 2.43, 95% CI [− 5.40, 0.28], *P* = 0.064] (see Supplementary Fig. 2 for scatterplot and see Supplementary Table 2 for all bootstrapped parameter estimates).

### Relation of covert experiential states to prior studies, location within the neural state space, and relationship between experiential states and target detection performance

**Figure 4:**
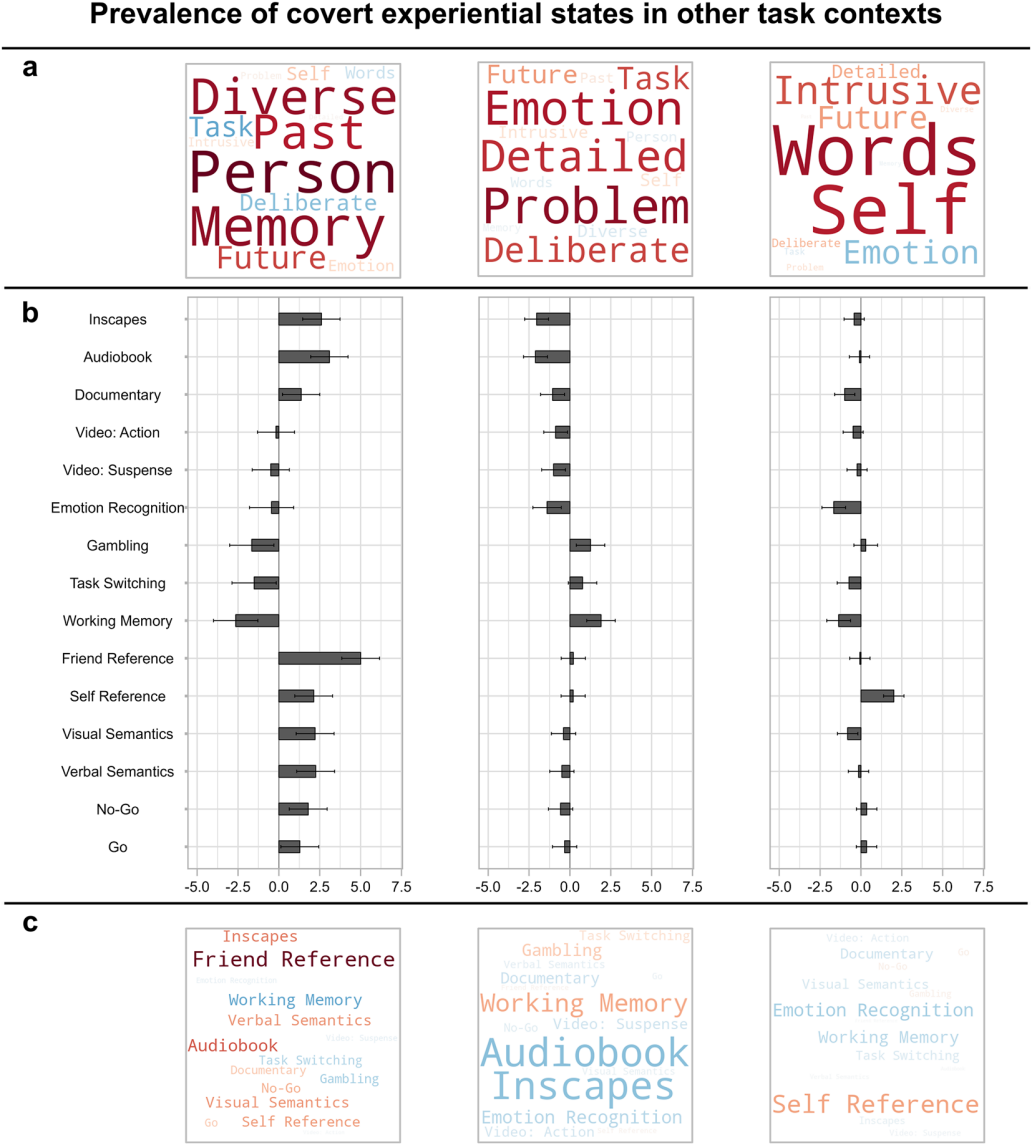
Prevalence of the three covert experiential states identified in the current study, during a simple sustained attention task, in other task contexts from an independent sample. (**a**) Word clouds representing the three experiential states identified by applying principal components analysis to the experience sampling data from the current study. The first describes patterns of off-task episodic social cognition, the second describes patterns of deliberate task focus, and the third describes patterns of verbal, self-relevant thought. Each item represents one of the 13 experience sampling items, the size of the word represents the magnitude of the component loading for that item, and the color represents the direction (warmer colors = positive loading, cooler colors = negative loading). We projected these three thought patterns, identified in the current study's data, onto experience sampling data from a previously published study^22^ by Konu et al. in which participants completed a wide range of conventional and naturalistic tasks in the behavioral laboratory, and described their experiences using the same battery of mDES questions (see “Materials and methods”). (**b**) Bar graphs showing the estimated marginal means (i.e., predicted means) of each thought pattern for each task context reported by Konu et al.^22^, estimated in a series of linear mixed models in which each thought pattern was the outcome variable and task context was the explanatory variable (N participants = 70, N observations = 2302; see “Materials and methods”). Y-axis shows the different task contexts and x-axis shows estimated marginal means. Each bar plot represents a different thought pattern, corresponding to the word cloud shown above in panel (**a**). Error bars represent 95% confidence intervals. (**c**) Word clouds representing the estimated marginal means for each task context for each thought pattern presented in panel (**b**). Each word represents one of the task contexts, the size of the word represents the magnitude of the estimated marginal mean, and the color represents the direction (warmer colors = positive, cooler colors = negative).

**Figure 5:**
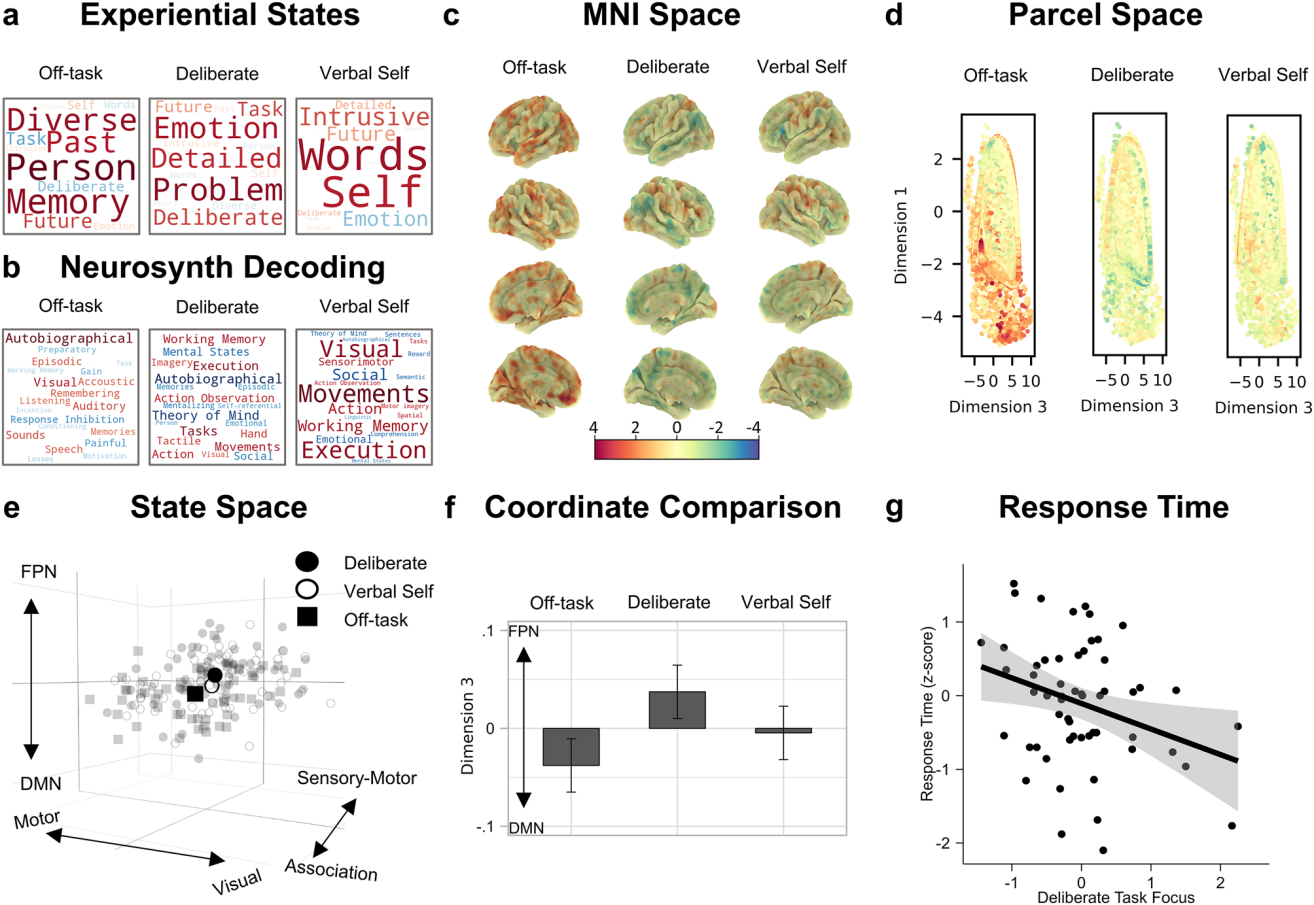
Location of experiential states in the neural state space and how self-reports of these states relate to task performance. (**a**) Word clouds representing the three experiential states identified by applying principal components analysis to the experience sampling data. The first describes patterns of off-task episodic social cognition, the second describes patterns of deliberate task focus, and the third describes patterns of verbal, self-relevant thought. Each item represents one of the 13 experience sampling items, the size of the word represents the magnitude of the component loading for that item, and the color represents the direction (warm = positive loading, cool = negative loading). (**b**) Word clouds representing the results from a meta-analysis using Neurosynth to decode the most likely terms used to describe the pattern of brain activity seen in the experiential maps. Size of the word represents the magnitude of the relationship, and the color represents the direction (warm = positive relationship, cool = negative relationship). (**c**) The (unthresholded) experiential group-level maps plotted on the cortical surface in MNI space. These three maps share a common color scale. (**d**) A scatter plot of the first and third dimensions of the state space (points = parcels), colored by the fMRI BOLD activity in the experiential brain maps shown in panel (**c**). (**e**) 3-d scatterplot showing where (1) individual experiential maps fall in the state space (smaller points) and (2) the average position of these maps across the sample (larger points). In this plot, each small point represents a whole-brain map for each experiential state for each participant (N observations = 171). (**f**) Bar graph showing the significant difference between off-task and deliberate task focus states along the default mode (DMN) to fronto-parietal (FPN) dimension of the state space. Y-axis represents the estimated marginal mean for each level of ‘experiential map'. Error bars represent 95% CIs (N observations = 171). (**g**) Scatterplot showing the relationship between target detection response time (z-scored seconds; y-axis) and the extent to which an individual reported deliberate task focus (x-axis) (N observations = 57).

Having established the position of the overt task states in the neural state space, we next examined how this common space also organized the position of the three covert experiential states identified by experience sampling. Principal components analysis (PCA) was applied to the mDES data, revealing three components, accounting for 47.41% of the total variance: (1) off-task episodic social cognition, (2) deliberate task focus, and (3) verbal, self-relevant thought (see “Materials and methods”). These components are represented as word clouds in Figs. 4a and 5a.

Before exploring the neural patterns associated with these covert experiential states, we first sought to contextualize these thought patterns, observed in our relatively non-demanding task, in the context of other task situations in which specific patterns of thought are likely to dominate. To this end, we projected the three thought patterns from the current data onto experience sampling data from a previously published study^22^ in which participants completed a wide range of conventional and naturalistic tasks in the behavioral laboratory, and described their experiences using the same battery of mDES questions. We then compared the prevalence of each thought pattern across these task situations in a series of linear mixed models (see “Materials and methods”). Reported P values in-text are unadjusted, unless specified in parentheses.

The mixed models indicated that the three thought patterns significantly varied according to task context [off-task thought: [*F*(14, 2204) = 40.18, *P* < 0.001]; deliberate task focus: [*F*(14, 2204) = 27.77, *P* < 0.001]; verbal self-relevant thought: [*F*(14, 2203) = 25.47, *P* < 0.001]]. The first pattern, off-task episodic social cognition, emerged most in the ‘friend-reference’ task in which participants were asked to rate a friend of choice along a set of personality adjectives (and that recruits key regions of the default mode network^40,41^), and, least in a spatial working memory task, which has a reliance on activity within the fronto-parietal network^42,43^. This highlights that the pattern of off-task thought seen in the current study is common in situations that encourage self-generated social cognition, and is relatively absent from tasks that require the maintenance of task-relevant information, a pattern that is consistent with situations in which this state emerges^8^. Second, we found that the pattern of deliberate task focus, in contrast, was most prominent in the spatial working memory task, gambling task, and task-switching, and was relatively absent from audiobook listening. The pattern of deliberate task focus observed in our study, therefore, emerges the most in situations that rely on executive control^44,45^, suggesting that it represents a covert state that is prominent when tasks rely on the maintenance of goals in working memory. Finally, the verbal, self-relevant thought pattern emerged most in the ‘self-reference’ task in which participants were asked to rate themselves along a set of personality adjectives^46,47^ and least in the emotion recognition task. The estimated marginal means for each thought pattern in each task are shown in a bar graph in Fig. 4b and also represented as word clouds in Fig. 4c.

Taken together, this analysis provides independent empirical support that in our study we identified two types of covert states. We found a pattern of off-task thought that was generally most common in situations conducive to self-generating episodic social information, a key feature of off-task thought^24,48^. Furthermore, we identified a pattern of detailed task focus that was generally prominent in situations reliant on executive control. This analysis highlights the laboratory situations in which we tend to see these patterns of thought, and so helps ground the covert states that are the focus of our study in a broader context.

Next, we examined how these covert states relate to patterns of brain activity. To this end, we performed a linear regression, at the individual-level, in which each individuals’ fMRI data during the six seconds prior to the experience-sampling probe was the outcome variable and the individual’s thought score on each of the thought patterns (1–3) was the explanatory variable (see “Materials and methods”). This produced, for each individual, a spatial map of how their brain activity was associated with their score on each of the three thought patterns identified via PCA.

Next, we calculated the pairwise correlations between each of these three ‘experiential’ maps and each of the five connectivity gradients, resulting in five coordinates per experiential map. The results of this process can be seen in Fig. 5e, and see Supplementary Fig. 1 for the distribution of these coordinates. To understand how these experiential maps are differentiable along the five dimensions of our neural state space, we compared the position (i.e., the coordinates) of these maps along each dimension in a series of linear mixed models (see “Materials and methods”). As before, in addition to traditional significance tests of main effects, we performed bootstrapping (n iterations = 1000) to calculate parameter estimates and their associated confidence intervals and P values (see Supplementary Table 3 for all bootstrapped estimates). Reported P values in-text are unadjusted, unless specified in parentheses.

The mixed models comparing the position of each experiential map along each dimension of the state space revealed that the position of the experiential maps differed significantly along dimension 3, which separates the default mode and fronto-parietal networks [*F*(2, 112) = 7.76, *P* < 0.001]. The off-task state fell towards the default mode end of this dimension [bootstrapped *b* = − 0.04, 95% CI [− 0.06, − 0.02], *P* < 0.001], while the deliberate task focus state fell towards the fronto-parietal end [bootstrapped *b* = 0.04, 95% CI [0.02, 0.06], *P* < 0.001]. Therefore, our results indicate that covert states of deliberate task focus are associated with patterns of brain activity emphasizing the fronto-parietal network rather than the default mode, whereas, covert off-task states tend to show the opposite pattern. In both cases, the maps were not only different from one another on the default mode to fronto-parietal dimension of the state space, but the distributions of both states fell on average outside the center of this dimension (see Fig. 5f). Our state space analysis suggests, therefore, that two of the patterns of thought, off-task thought and deliberate task focus, correspond to brain states that are dominated by the default mode and fronto-parietal networks respectively. Moreover, the Neurosynth decoding associated with these maps revealed patterns that emphasized memories (off-task thought) and working memory/task execution (deliberate task focus) (see Fig. 5b). Importantly these meta-analytic patterns are broadly consistent with our prior analysis that mapped the current mDES patterns across a range of task contexts in an independent sample (see Fig. 4).

Having established how the patterns of ongoing thought relate to whole-brain patterns of brain activity, we next examined how the covert states’ positions in the neural state space related to target detection performance during the task. To this end, we performed a multiple regression in which the covert state maps’ coordinates were the explanatory variables, and response time was the outcome variable (see “Materials and methods”). This analysis indicated that the position of the covert states in state space did not significantly relate to response time (see Supplementary Table 4 for all bootstrapped parameter estimates). Finally, we conducted a complementary analysis to identify how individual variation along each of the three patterns of thought derived from mDES was associated with response time. This analysis indicated that self-reports of deliberate task focus were associated with faster response times during target detection [*F*(1,50) = 5.51*, P* = 0.023; bootstrapped *b* = − 0.34, 95% CI [− 0.60, − 0.01], *P* = 0.046]. This suggests that patterns of covert deliberate thought may support better task performance (see Fig. 5g for scatterplot and Supplementary Table 5 for all bootstrapped parameter estimates).

## Supplementary analyses

### Network averages of overt task states and covert experiential states

Finally, we also performed a supplementary analysis in which we compared the mean level of activity in each of the Yeo-7 networks^36^ between each overt task state using a linear mixed model in which the average activity level was the outcome variable and ‘state’ (which overt task state; 2 levels) and ‘network’ (which Yeo-7 network; 7 levels) were the explanatory variables as well as their two-way interaction. In this model, the main effects are of no theoretical interest but probing the two-way interaction between ‘state’ and ‘network’ reveals how mean activation differs between states in each network. We also ran the equivalent model for the covert experiential states (see Supplementary Methods).

For the overt states, there was a significant two-way interaction between ‘state’ and ‘network’ [F(1,728) = 27.72, P < 0.001]. Pairwise comparisons indicated that compared to vigilance states, target detection states had significantly higher mean activation in the dorsal attention [b = − 1.20, 95% CI [− 1.58, − 0.82], t(728) =  − 8.47, adjusted P < 0.001], ventral attention [b = − 0.88, 95% CI [− 1.26, − 0.50], t(728) =  − 6.26, adjusted P < 0.001] and fronto-parietal networks [b = − 0.70, 95% CI [− 1.08, − 0.32], t(728) =  − 4.96, adjusted P < 0.001], and, significantly lower mean activation in the limbic [b = 0.54, 95% CI [0.16, 0.92], t(728) = 3.84, adjusted P < 0.001] and default mode networks [b = 0.75, 95% CI [0.37, 1.13], t(728) = 5.32, adjusted P < 0.001] (P values Bonferroni-corrected for 7 comparisons). For the covert states, the two-way interaction between ‘state’ and ‘network’ was not significant [F(1,1120) = 0.76, P = 0.690]. For all estimated marginal means for each network and state combination emerging from these models, see Supplementary Tables 6 and 7. The mean activation levels in each network for the group-level brain maps are also represented as radar plots in Fig. 2b,c.

## Discussion

Our study set out to examine whether experience sampling can be used to provide insights into covert task-relevant states that are hypothesized to play a role in organizing task behavior. To address this question, we used a simple sustained attention task in which participants detected visually presented targets (to provide an index of overt behavioral states) and experience sampling (to provide indices of covert cognitive states). To better understand the patterns of thought identified by experience sampling, we projected the patterns from our current data set onto experience sampling data from an independent behavioral study in which participants performed a range of different tasks with varying demands^22^ to understand the situations in which these thought patterns dominate. To allow the mapping between overt and covert states and whole-brain patterns of brain activity, we used a neural state space derived from dimensions of brain function variation^12^ calculated from the resting state data of the Human Connectome Project^35^. These dimensions characterize five different types of brain organization: (1) the distinction between sensory-motor and association cortex, (2) the distinction between motor and visual cortex, (3) the distinction between the default mode and fronto-parietal networks in association cortex, (4) the distinction between the dorsal attention network and regions of visual cortex and ventral attention network, and (5) the distinction between the visual cortex and ventral attention network (see Fig. 1, second panel, for spatial brain maps representing each of these dimensions and see Fig. 2a for radar plots showing their network configuration).

Our study shows that experience sampling is sensitive to at least two distinct forms of covert state. Consistent with a wide range of prior studies (e.g.,^10,14,22^), we established a state of off-task social episodic cognition, that was prominent in task situations where individuals are asked to imagine other people, and was absent from demanding tasks like spatial working memory (see Fig. 4). This pattern of thought was related to patterns of whole-brain activity in which the default mode network was more prominent than the fronto-parietal network (see Fig. 5, panel f), and meta-analytic decoding highlighted similarities with prior studies on autobiographical memory (see Fig. 5, panel b). At the same time, consistent with prior work^22,24,25,49^, we found a pattern of deliberate task focus that was most prominent in task situations reliant on executive control (see Fig. 4). This state was associated with whole-brain patterns in which the fronto-parietal network was more prominent than the default mode network (see Fig. 5, panel f), and meta-analytic decoding suggested that this pattern was seen in situations of working memory or task execution, rather than autobiographical memory or theory of mind (see Fig. 5, panel b). Furthermore, in our study, self-reported deliberate task focus was associated with faster target detection, providing behavioral evidence that the covert state identified in the current study was linked to beneficial, rather than detrimental task performance (see Fig. 5, panel g). In this way, the current study demonstrates that experience sampling is capable of reliably identifying covert states with different features, and so extends the utility of this method beyond understanding introspective states (like mind-wandering^1,50^) to encompass covert states that help support more effective task performance.

As well as establishing the value of experience sampling in understanding task-relevant states, our study provides important insights regarding the mapping between cognition and brain activity. Our study suggests that the primary difference in whole-brain activity between the two covert states was in the balance of activity between the two large-scale systems in association cortex described by the third dimension: the fronto-parietal and default mode networks. It is interesting to note that both deliberate task focus, and off-task episodic thought may involve the maintenance of stimulus-independent information in awareness. At the level of self-reports, off-task thoughts were characterized by features related to different temporal periods (future and past), while deliberate task focus was associated with a detailed focus on task-relevant information even though the experience sampling probes occurred in moments when there was no immediate opportunity for action (during vigilance periods). Furthermore, by comparison within an independent data set, we found that patterns of detailed task focus were most dominant when participants must maintain task rules in attention (i.e., task switching) or when they must maintain task relevant information in working memory. In contrast, patterns of off-task social episodic thought were most common in a task that involves mentally simulating a familiar other.

Although our study demonstrates the value of experience sampling in understanding covert task-relevant states, it nonetheless leaves several questions unanswered. First, since we focused only on one task context, it remains unclear how the current findings generalize to situations with different features. In order to fully understand the role association cortex plays in behavior, it is likely to be particularly important to explore task situations where information from memory is important for guiding actions^41,51,52^ and in tasks where performance depends on integration between memory and different types of sensory input (e.g., movie watching or semantic decision making). In the future, therefore, it will be valuable to employ our state space approach across a wider range of task contexts. This is particularly important given an emerging body of work highlighting the importance of considering the context in which covert experiences emerge when investigating their psychological and neural correlates^8,22,23,53,54^. Second, since our study focused only on a single scanning session, it will be important for future work to evaluate the consistency and reliability of findings, within and across individuals and over time. For example, future work could use more intensive scanning procedures like those employed in the Midnight Scan Club^55^ to evaluate the stability of brain states’ locations in the state space.

Third, although we identified a relationship between deliberate task focus and better task performance, the significance of this relationship would likely be improved if we could identify situations that maximize the beneficial value of deliberate thought to performance. For example, in a large behavioral sample, Turnbull et al.^56^ demonstrated that off-task thought was more detrimental to performance in a working memory task than in a choice reaction time task. It could be important in the future to understand how variations in deliberate task focus impact on performance across a range of different task conditions to better understand how this thought pattern supports better performance. Finally, although the state space approach is useful for identifying coarse similarities and differences in whole-brain patterns between states, it may be limited in its ability to identify more fine-grained details. Although this approach is likely to be insufficient to detail modular functions within regions, as our study highlights, it provides a simple way to understand commonalities in brain organization between different types of state and thus is a powerful method to investigate domain general perspectives on how brain organization gives rise to cognition and behavior.

We close by considering one of the most intriguing questions to emerge from our study: the possibility that association cortex plays a general role in allowing ongoing thought to draw on stimulus-independent information, either to complement ongoing actions, processes linked to executive control^57^, or, in the service of explicitly simulating situations from memory (a phenomenon often referred to as mental time travel^26,32,58–62^ or as scene construction^63^). For example, our meta-analysis of the brain activity seen during covert states of deliberate task focus highlighted links with situations when task relevant information is maintained in working memory, a pattern also supported by evidence that this pattern of thought was most prevalent during a visuo-spatial working memory task, gambling task, and task switching task in an independent behavioral study^22^. In contrast, off-task thought was associated with experiential features linked to memory and different time periods (the future and the past), rather than the task at hand. Meta-analytic decoding of the associated brain maps highlighted autobiographical memory, among other features, as important. It is commonly assumed that states like mental time travel^64–66^ help organize behavior over time^60,61^ and may be linked to the default mode network through its role in the ability to replay information from memory^67^. More generally, this perspective is consistent with the alignment between the default mode network and features of declarative long-term memory such as semantic or episodic memory^68^.

In the future, therefore, it will be important to understand how different systems within association cortex, which clearly contribute to cognitive functions in different situations, may do so through a reliance on stimulus-independent features of cognition to help organize behavior (see^37^ for further discussion). Such a perspective draws support from contemporary views on the topology of brain organization that suggests regions making up both the fronto-parietal and default mode networks are located in regions of the cortex that are maximally distant from the systems involved in action and perception^12^. The location of association cortex at the maximal distance from sensory-motor systems may provide a mechanism through which brain activity in these regions can be more distinct from activity in regions that respond to input describing the immediate environment (for a discussion see:^37^). Our data, therefore, is consistent with contemporary perspectives on association cortex that suggest that the distance along the cortical mantle from the brain’s input–output systems may be important for helping support patterns of brain activity that are less directly related to information in the immediate environment. More recently, Leech et al.^69^, found that both the fronto-parietal and default mode networks exhibit more rapid functional change across the cortical mantle than seen in primary systems, a pattern that may be attributable to the relatively low levels of myelin seen within association cortex. It is possible, therefore, that we can improve our understanding of both similarities and differences between the fronto-parietal and default mode networks through a better understanding of the underlying structure associated with the observed patterns of activation^70^.

## Materials and methods

In the following “Materials and methods”section, the details for the participant information, task paradigm, experience sampling, task procedure, fMRI acquisition and preprocessing, principal components analysis, and FSL-based fMRI analysis are the same as those described in Konu et al.^11^, with minor rewording in places for clarity (material originally published under a Creative Commons License: CC BY-NC-ND 4.0).

### Participants

One hundred and seven participants took part in this study. Ninety-one participants participated in a behavioral session (67 females; mean age 23.38 years, standard deviation 4.53 years, age range 19–40 years). Sixty-two participants participated in the scanning session (41 females; mean age 23.29 years; standard deviation 4.51 years, age range 18–39 years). After excluding 5 participants, 57 remained for fMRI data analysis (due to technical difficulties or excess movement). Forty-six participants participated in both the behavioral and scanning session. All participants had normal/corrected vision and had no history of psychiatric or neurological illness. All scanning participants were right-handed. This cohort was acquired from the undergraduate and postgraduate student population at the University of York. The study was approved by the local ethics committee at the York Neuroimaging Centre and University of York Psychology Department and all research was performed in accordance with relevant guidelines and regulations. All volunteers provided informed written consent and received monetary compensation or course credit for their participation. These details are the same as those described in Konu et al.^11^.

### Task paradigm

Participants were instructed to attend to the center of the screen while they were presented with a sequence of ‘non-target’ and ‘target’ stimuli, to which they responded only to target stimuli (mean stimulus presentation duration: 1000 ​ms). Therefore, this task comprised of ‘vigilance’ periods, in which non-target stimuli were presented, and ‘target detection’ periods, in which target stimuli were presented, requiring a button push. A single run of the task was 13 ​min and contained eight instances of experience sampling probes. All experience sampling probes were presented in vigilance periods. For each experience sampling probe, participants rated each experience sampling item once (see the next section for details of the experience sampling technique). Each of the 13 experience sampling items were presented for a maximum of 4 ​s on the screen—based on the average response time from previous studies—followed by a 500 ​ms fixation cross. The remainder of the time was allocated to two kinds of experimental trials: target and non-target. In target trials, a green circle was randomly presented (20% of the experiment trials) and participants were required to make a response (a single key or button press). In non-target trials, a red octagon was presented (80% of the experiment trials) and no behavioral response was required. An experimental trial was fixed at 3000 ​ms. The inter-stimulus-intervals (ISI) consisted of a fixation cross and was jittered (1500–2500 ​ms). The stimulus was presented on screen for 500–1500 ​ms until a response was made. Once a response was captured, a fixation cross appeared on the screen for the remaining time. We chose this task because prior studies (e.g.,^20^) have established that in tasks with relatively low demands, a balance of both task-focused states and self-generated states can emerge, providing the opportunity to realize both task-relevant and task-irrelevant covert states. The task paradigm is presented schematically in the top panel of Fig. 1. In the scanner, participants completed three runs of the task, whereas, in the behavioral session they completed one run of the task. Written instructions were presented at the start of each run. These details are the same as those described in Konu et al.^11^.

### Multidimensional experience sampling (mDES)

Participants’ ongoing thought throughout the task was measured using a technique known as multidimensional experience sampling (mDES)^21^. When an mDES probe occurred, participants were first asked how much their thoughts were focused on the task, followed by 12 randomly shuffled items about the content and form of their thoughts (see Supplementary Table 8 for all mDES items). All items were rated on a 1–10 continuous scale (see top panel of Fig. 1 for an illustration). Within one run of the task, participants completed eight sets of mDES probes, yielding a total of eight probes per individual in the behavioral session and 24 probes per individual in the scanning session. In the scanning session, two (out of 57) participants had one run dropped due to technical issues, leaving them with 16 probes overall. These details are the same as those described in Konu et al.^11^.

### Procedure

In the behavioral session, participants completed a single 13-min run of the task with mDES. In the scanning session, participants completed three, 13-min functional runs of the task with mDES while undergoing fMRI. The scanner session took around 1 ​h and 15 ​min, of which the task took ~ 45 ​min, and this was separated into three blocks. These details are the same as those described in Konu et al.^11^.

### fMRI acquisition

All MRI scanning was carried out at the York Neuroimaging Centre. Structural and functional scans were acquired using a Siemens Prisma 3 T MRI Scanner with a 64-channel phased-array head coil. Structural data were acquired using a T1-weighted (MPRAGE) whole-brain scan (TR ​ = ​2300 ​ms, TE ​ = ​2.26 ​ms, flip angle ​ = ​8°, matrix size ​ = ​256 × 256, 176 slices, voxel size ​ = ​1 × 1 ​× ​1 ​mm). Functional data were collected using a gradient-echo EPI sequence with 54 bottom-up interleaved axial slices (TR ​ = ​3000 ​ms, TE ​ = ​30 ​ms, flip-angle ​ = ​80°, matrix size ​ = ​80 × 80, voxel size ​ = ​3 × 3 ​ × ​3 ​mm, 267 ​vol) covering the whole brain. These details are the same as those described in Konu et al.^11^.

### fMRI data pre-processing

Functional and structural data were pre-processed and analyzed using FMRIB’s Software Library (FSL, version 5.0.1, http://fsl.fmrib.ox.ac.uk/fsl/fslwiki/FEAT/). Individual T1-weighted structural images were extracted using BET (Brain Extraction Tool). Functional data were pre-processed and analyzed using the FMRI Expert analysis Tool (FEAT). Individual participant analysis involved motion correction using MCFLIRT and slice-timing correction using Fourier space time-series phase-shifting. In the current study, to control for individual’s movement during the scanning period in our inferential analyses, we also calculated each individual’s mean movement across all three runs using the MCFLIRT output to include as a nuisance regressor in subsequent analyses (prefiltered_func_data_mcf_abs_mean.rms). After co-registration to the structural images, individual functional images were linearly registered to the MNI-152 template using FMRIB’s Linear Image Registration Tool (FLIRT). Registration from high resolution structural to standard space was then further refined using FNIRT nonlinear registration. Functional images were spatially smoothed using a Gaussian kernel of FWHM 6 ​mm, underwent grand-mean intensity normalization of the entire four-dimensional dataset by a single multiplicative factor, and had high pass temporal filtering (Gaussian-weighted least-squares straight line fitting, with sigma ​ = ​50 s). These details are the same as those described in Konu et al.^11^.

### Principal component analysis

Analysis of the mDES data was carried out in SPSS (Version 25, 2019). Principal component analysis (PCA) was applied to the scores from the 13 experience-sampling items comprising the probes for each participant. PCA was applied at the probe level in the same manner as in our prior studies e.g.,^21–23,29,71–73^. Specifically, we concatenated the responses of each participant for each probe into a single matrix—in which each column was an experience sampling item (13 items), and each row was an mDES observation—and employed a PCA with varimax rotation. We performed this analysis separately for each session (behavioral and scanning) in order to examine the similarity in the solutions produced across each situation (see Supplementary Fig. 3). For the scanning session, 62 participants data was included in the PCA, and each participant had three runs of eight probes (24 total), with the exception of four participants who only had 16 probes each, meaning that the PCA was applied to a 1456 × 13 matrix. For the behavioral session, 91 participants data was included in the PCA, and each participant had one run of eight probes, meaning that the PCA was applied to a 728 × 13 matrix. These details are the same as those described in Konu et al.^11^. This analysis resulted in a set of three PCA scores for each observation, indicating the prevalence of each PCA component identified for that observation (via the computation of the dot product between the component loadings of each component and the experience sampling items of that observation). In the current study, we calculated each individual’s mean score, across runs, for each PCA component identified for inclusion in inferential analyses. Intraclass correlation (ICC) analyses indicated moderate consistency of component 1 (0.68), and good consistency of component 2 (0.77) and component 3 (0.75) across the three runs.

### Projecting thought patterns identified via principal components analysis onto an independent dataset

In order to contextualize the three thought patterns identified by applying PCA to the current study’s experience sampling data, we projected the thought patterns onto experience sampling data from a previously published dataset by Konu et al.^22^ in which participants completed a wide range of conventional and naturalistic tasks across a two-day study (see Fig. 4). In total, there were 70 participants in this dataset (60 females; mean age 20.60 years; standard deviation 2.10 years, age range 18–34 years). For full methodological details of this study, see Konu et al.^22^. Prior to projection, the independent experience sampling data was scaled according to the mean and standard deviation of the experience sampling data of the current study (i.e., the dataset the PCA was trained on). The projection follows the same process that is used to calculate PCA scores in a regular PCA analysis, and simply involves computing the dot product between the component loadings of each thought pattern (identified in the current study) and each observation of experience sampling data (from the independent dataset), resulting in three scores per observation, indicating the prevalence of each thought pattern for that observation (N observations = 2302). These projected PCA scores were used as outcome variables in a series of linear mixed models (see below) to examine how the three thought patterns vary across a wide range of task contexts.

### fMRI analysis

#### Creating brain maps for covert experiential states and overt task states

Task-based analyses were carried out using FSL. A model was set up including six explanatory variables (EVs). EVs 1 and 2 modeled ‘vigilance’ and 'target detection’ periods. EV 3 modeled activity 6 s prior to each mDES probe. Finally, EVs 4, 5, and 6 modeled the three thought components identified through PCA, with a time period of 6 s prior to the mDES probes and the scores for the relevant component as a parametric regressor. EVs were mean-centered within each run and no thresholding was applied to the EVs. Standard and extended motion parameters were included as confounds. This was convolved with a hemodynamic response function using FSL’s gamma function. We chose to use the same 6 s interval as used in Turnbull et al.^29^. Contrasts were included to assess brain activity that related to each of the two task events (vigilance and target detection) and that related to each component of thought during the 6 s period prior to the probe. The three runs were included in a fixed-level analysis to average across the activity within an individual. The averaged-run individual-level unthresholded (z-stat) contrast maps were used in the state space analyses described below. Group-level analyses followed best practice^74^. Specifically, we used FLAME, as implemented by FSL. These details are the same as those described in Konu et al.^11^. Figures 3a and 5c show the unthresholded group-level maps in MNI space while Figs. 3b and 5d show scatterplots representing how the BOLD activity in the unthresholded group-level maps’ parcels is distributed along the state space dimensions. The unthresholded group-level maps were used in the Neurosynth analysis described in the next section, and radar plots representing the mean activation levels in each of the Yeo-7 networks^36^ for these group-level maps are shown in Fig. 2 (panel a and b).

#### Neurosynth decoding of covert experiential and overt task spatial maps

We used Neurosynth’s online meta-analytical decoder^75^ to identify cognitive and psychological terms most strongly associated with our (unthresholded) group-level neural maps in the available literature (five maps: vigilance, target detection, off-task thought, deliberate thought, and verbal, self-relevant thought). Using reverse inference analysis, the decoder identifies terms that are the most strongly associated with the patterns of neural activity in each unthresholded map. It does this by comparing the patterns of activity in the input map (in this case, overt and covert group-level maps) to patterns of activity in the brain maps available in the Neurosynth database. This results in a set of cognitive, anatomical, and psychological terms that are most likely to be positively or negatively associated with the patterns of activity in the input map. To visualize the results of this analysis as word clouds, we selected the top ten positive and top ten negative cognitive and psychological terms associated with each map, retaining only the first term in instances of duplicates (e.g., ‘autobiographical’ and ‘autobiographical memory’) and excluding terms related to anatomy instead of function (e.g., ‘occipital’). These word clouds are shown in Fig. 3c (overt states) and Fig. 5b (covert states).

#### Connectivity gradients used to construct the 5-d neural state space

The five connectivity gradients used in the current study were generated by Margulies et al.^12^ and are openly available via Neurovault (cortical and subcortical): https://identifiers.org/neurovault.collection:1598. These gradients were generated by applying a non-linear dimension reduction technique (diffusion embedding) to the averaged functional connectivity matrix of the Human Connectome Project (HCP) data^35^. These gradients explain whole-brain connectivity variance in descending order, such that the first gradient explains the most variance in the whole-brain connectivity data, the second explains the second most variance, and so on. Along each gradient, brain regions with similar connectivity profiles (to the rest of the brain) fall close together, and have similar ‘gradient values’, while regions with more distinct connectivity profiles fall further apart, and have more dissimilar ‘gradient values’^13^. This analysis, therefore, results in a spatial map for each gradient identified in which each parcel contains a ‘gradient value’ (see Fig. 1). The first five gradients explain approximately 60% of the connectivity variance and prior studies have highlighted that the first three gradients relate to important features of cognition^14,52,76^. The first gradient describes the difference between sensory-motor regions and association cortex. The second gradient separates motor and visual systems. The third gradient describes the difference between the default mode and the fronto-parietal networks. The fourth gradient describes the difference between the dorsal attention network and regions of the visual cortex and ventral attention network. Finally, the fifth gradient describes the difference between the visual cortex and ventral attention network (see Fig. 2a for radar plots representing the mean gradient values in each of the Yeo-7 networks^36^ for each gradient). We use these five gradients to construct the 5-d neural state space (see below).

#### Locating overt task states and covert experiential states in the neural state space

To locate overt task states and covert experiential states in the neural state space, we calculated the pairwise spatial correlations (Spearman rank) between each individual’s covert and overt brain maps and each of the first five connectivity gradients described in Margulies et al.^12^. The code for this process is openly available on GitHub: https://github.com/willstrawson/StateSpace. Therefore, for each individual, this resulted in five correlation values for each brain map, indicating where that brain map falls along each dimension of the neural state space. These correlation values act as ‘coordinates’ in the 5-d neural state space (see Fig. 1 for a 3-d scatterplot showing the coordinates for each state along the first three dimensions).

### Linear mixed models

Linear mixed models (LMMs) were fitted by restricted maximum-likelihood estimation in R [4.1.1^77^] using the lme4 package [1.1.31^78^]. We used the lmerTest package [3.1.3^79^] to obtain *P* values for the F-tests returned by the lme4 package. For each set of models, the alpha level for each F-statistic was set based on 0.05 divided by the number of models (i.e., Bonferroni-corrected alpha level). With the exception of pairwise comparisons, where adjusted P values are highlighted in parentheses, the reported P values are unadjusted. Degrees of freedom were calculated using Satterthwaite approximation and for F-tests, type 3 sum of squares was used. Contrasts were set to “contr.sum,” meaning that the intercept of each model corresponds to the grand mean of all conditions and that when a factor has two levels, the parameter estimate is equal to half of the difference between the two levels^80^. Estimated marginal means (shown in Figs. 3e, 4b, and 5f) were calculated using the emmeans package [1.8.3^81^]. Across all models, to account for multiple observations per participant, ‘participant’ was included as a random intercept. Finally, to establish the robustness of our results, we used the easystats package [0.6.0^82^] to obtain bootstrapped parameter estimates (n iterations = 1000) and their associated confidence intervals and *P* values.

### Comparing the location of overt task states in the neural state space

We ran five LMMs—one with each dimension coordinate as the outcome variable and “task map” as the explanatory variable (two levels: vigilance and target detection). Age, gender, and mean movement were included as nuisance covariates. In total, 57 participants were included in these models. These models allowed us to investigate how the location of the two overt task states (vigilance and target detection) differed along each dimension of the neural state space.

Example model formula: lmer(Dimension Coordinate X ~ Task Map + Age + Gender + Mean Movement + (1|Participant)).

### Comparing the prevalence of each covert experiential state across task contexts in an independent sample

We ran three LMMs—one with each (projected) thought pattern as the outcome variable and “task context” as the explanatory variable (15 levels). In line with the mixed models performed in Konu and colleagues’ original study^22^, probe order (indicating which probe number within each task), day number (indicating which day of testing), and order (of task session, counterbalanced across participants) were included as nuisance covariates. See the original study^22^ for full procedural details. In total, 70 participants (2302 observations) were included in these models. These models allowed us to investigate how the thought patterns identified in the current study emerge across a range of task contexts in an independent sample.

Example model formula: lmer(Thought pattern X ~ Task Context + Probe Order + Order + Day + (1|Participant)).

### Comparing the location of covert experiential states in the neural state space

We ran five LMMs—one with each dimension coordinate as the outcome variable and “experiential map” as the explanatory variable (three levels: off-task thought, deliberate thought, and verbal, self-relevant thought). Age, gender, and mean movement were included as nuisance covariates. In total, 57 participants were included in these models. These models allowed us to investigate how the location of the three covert experiential states (off-task, deliberate, and verbal, self-relevant thought) differed along each dimension of the neural state space.

Example model formula: lmer(Dimension Coordinate X ~ Experiential Map + Age + Gender + Mean Movement + (1|Participant)).

### Multiple regressions

Multiple regressions were fitted by ordinal least squares (OLS) in R [4.1.1^77^] using the stats package. We used the rstatix package [0.7^83^] to obtain F-test statistics and associated P values. For each regression model, the alpha level for each F-statistic was 0.05. For F-tests, type 3 sum of squares was chosen and contrasts were set to “contr.sum,” meaning that the intercept of each model corresponds to the grand mean of all conditions and that when a factor has two levels, the parameter estimate is equal to half of the difference between the two levels^80^. To establish the robustness of our results, we used the easystats package [0.6.0^82^] to obtain bootstrapped parameter estimates (n iterations = 1000) and their associated confidence intervals and *P* values. Across all models, age, gender, and mean movement were included as nuisance covariates. Finally, in these reaction time models, cases exhibiting a z-scored response time greater than 2.5 were considered outliers, and the z-scores of these outliers were set to zero to mitigate their influence on the results. Using this approach, two cases were considered outliers.

### Examining how the location of overt task states in the neural state space relate to target detection reaction time

We ran a multiple regression in which response time (z-scored) was the outcome variable, and the five coordinates for each of the two overt task brain maps (vigilance and target detection) were the explanatory variables (10 in total). In total, 57 participants were included in these models. This regression allowed us to investigate whether there is a correspondence between the location of overt task states within the neural state space and target detection performance.

Example model formula: lm(Z-scored Response Time ~ Target Detection Coordinate along Dimension 1 + Target Detection Coordinate along Dimension 2 + Target Detection Coordinate along Dimension 3 + Target Detection Coordinate along Dimension 4 + Target Detection Coordinate along Dimension 5 + Vigilance Coordinate along Dimension 1 + Vigilance Coordinate along Dimension 2 + Vigilance Coordinate along Dimension 3 + Vigilance Coordinate along Dimension 4 + Vigilance Coordinate along Dimension 5 + Age + Gender + Mean Movement).

### Examining how the location of covert experiential states in the neural state space relate to target detection reaction time

We ran a multiple regression in which response time (z-scored) was the outcome variable, and the five coordinates for each of the three covert experiential brain maps (off-task thought, deliberate task focus, and verbal, self-relevant thought) were the explanatory variables (15 in total). In total, 57 participants were included in these models. This regression allowed us to investigate whether there is a correspondence between the location of covert experiential states within the neural state space and target detection performance.

Example model formula: lm(Z-scored Response Time ~ Off-task Thought Coordinate along Dimension 1 + Off-task Thought Coordinate along Dimension 2 + Off-task Thought Coordinate along Dimension 3 + Off-task Thought Coordinate along Dimension 4 + Off-task Thought Coordinate along Dimension 5 + Deliberate Thought Coordinate along Dimension 1 + Deliberate Thought Coordinate along Dimension 2 + Deliberate Thought Coordinate along Dimension 3 + Deliberate Thought Coordinate along Dimension 4 + Deliberate Thought Coordinate along Dimension 5 + Verbal Self-relevant Thought Coordinate along Dimension 1 + Verbal Self-relevant Thought Coordinate along Dimension 2 + Verbal Self-relevant Thought Coordinate along Dimension 3 + Verbal Self-relevant Thought Coordinate along Dimension 4 + Verbal Self-relevant Thought Coordinate along Dimension 5 + Age + Gender + Mean Movement).

### Examining how experiential reports of covert states relate to target detection reaction time

We ran a multiple regression in which response time (z-scored) was the outcome variable, and individual’s mean PCA scores for each of the three thought patterns (off-task thought, deliberate task focus, and verbal, self-relevant thought) were the explanatory variables. In total, 57 participants were included in these models. This model allowed us to investigate whether there is a correspondence between individual’s reported covert experiences and their target detection performance.

Example model formula: lm(Z-scored Response Time ~ Off-task Thought + Deliberate Thought + Verbal Self-relevant Thought + Age + Gender + Mean Movement).

### Supplementary Information

Supplementary Information.

## Data Availability

Ethical approval conditions and European Research Council (ERC) grant stipulations do not permit the public sharing of raw data. However, anonymized Multidimensional Experience Sampling data, state space coordinates, and network averages are publicly available via Mendeley: https://doi.org/10.17632/mx76fvdm3v.2. In addition, all group-level unthresholded brain maps presented in the figures are available via NeuroVault: https://identifiers.org/neurovault.collection:13520. The code for the task paradigm is publicly available at: https://vcs.ynic.york.ac.uk/hw1012/go_nogo_experience_sampling/tree/master/. All code used in the current analysis and preparation of figures is publicly available via GitHub at: https://github.com/Bronte-Mckeown/mDES_States.
