# Supplementary material for: Experience sampling reveals the role that covert goal states play in task-relevant behavior
Source: Sci Rep. 2023 Dec 7;13:21710. doi: 10.1038/s41598-023-48857-0 (PMC10709616; doi:10.1038/s41598-023-48857-0)
Supplement: Supplementary file 1 — Supplementary Information. [file 41598_2023_48857_MOESM1_ESM.docx]

**Supporting Information for**

**Experience sampling reveals the role that covert goal states play in task-relevant behavior.**

Brontë Mckeown *^1^, Will H. Strawson ^2^, Meichao Zhang ^3, 4^, Adam Turnbull ^5, 6^, Delali Konu ^7^, Theodoros Karapanagiotidis ^8^, Hao-Ting Wang ^9^, Robert Leech ^10^, Ting Xu ^11^, Samyogita Hardikar ^12^, Boris Bernhardt ^13^, Daniel Margulies ^14^, Elizabeth Jefferies ^15^, Jeffrey Wammes ^1^ & Jonathan Smallwood ^1^

Corresponding author: Brontë Mckeown; [bronte.mckeown@queensu.ca](mailto:bronte.mckeown@queensu.ca)

**This file includes:**

Supplementary Methods

Supplementary Figures 1 to 3

Supplementary Tables 1 to 8

Supplementary Methods

Linear mixed models

Linear Mixed Models (LMMs) were fitted by restricted maximum-likelihood estimation in R [4.1.1 ^1^] using the lme4 package [1.1.31 ^2^]. We used the lmerTest package [3.1.3 ^3^] to obtain *P* values for the F-tests returned by the lme4 package. The alpha level for each F-statistic was set based on 0.05 divided by the number of models (i.e., Bonferroni-corrected alpha level; 0.05/2). Degrees of freedom were calculated using Satterthwaite approximation and for F-tests, type 3 sum of squares was used. Contrasts were set to “contr.sum,” meaning that the intercept of each model corresponds to the grand mean of all conditions and that when a factor has two levels, the parameter estimate is equal to half of the difference between the two levels ^4^. Estimated marginal means (shown in Figure 2E and 3F) were calculated using the emmeans package [1.8.3 ^5^]. Across all models, to account for multiple observations per participant, ‘participant’ was included as a random intercept.

Comparing average activation levels in each of the Yeo-7 Networks between overt task states.

We ran one LMM—with average activation level as the outcome variable and ‘state’ (which overt task state; 2 levels) and ‘network’ (which Yeo-7 network; 7 levels) were the explanatory variables as well as their two-way interaction. In total, 57 participants were included in these models. Age, gender, and mean movement were included as nuisance regressors.

Example model formula: lmer(Average Activation ~ State * Network + Age + Gender + Mean Movement + (1|Participant))

Comparing average activation levels in each of the Yeo-7 Networks between covert experiential states.

We ran one LMM—with average activation level as the outcome variable and ‘state’ (which covert experiential state; 3 levels) and ‘network’ (which Yeo-7 network; 7 levels) were the explanatory variables as well as their two-way interaction. In total, 57 participants were included in these models. Age, gender, and mean movement were included as nuisance regressors.

Example model formula: lmer(Average Activation ~ State * Network + Age + Gender + Mean Movement + (1|Participant))


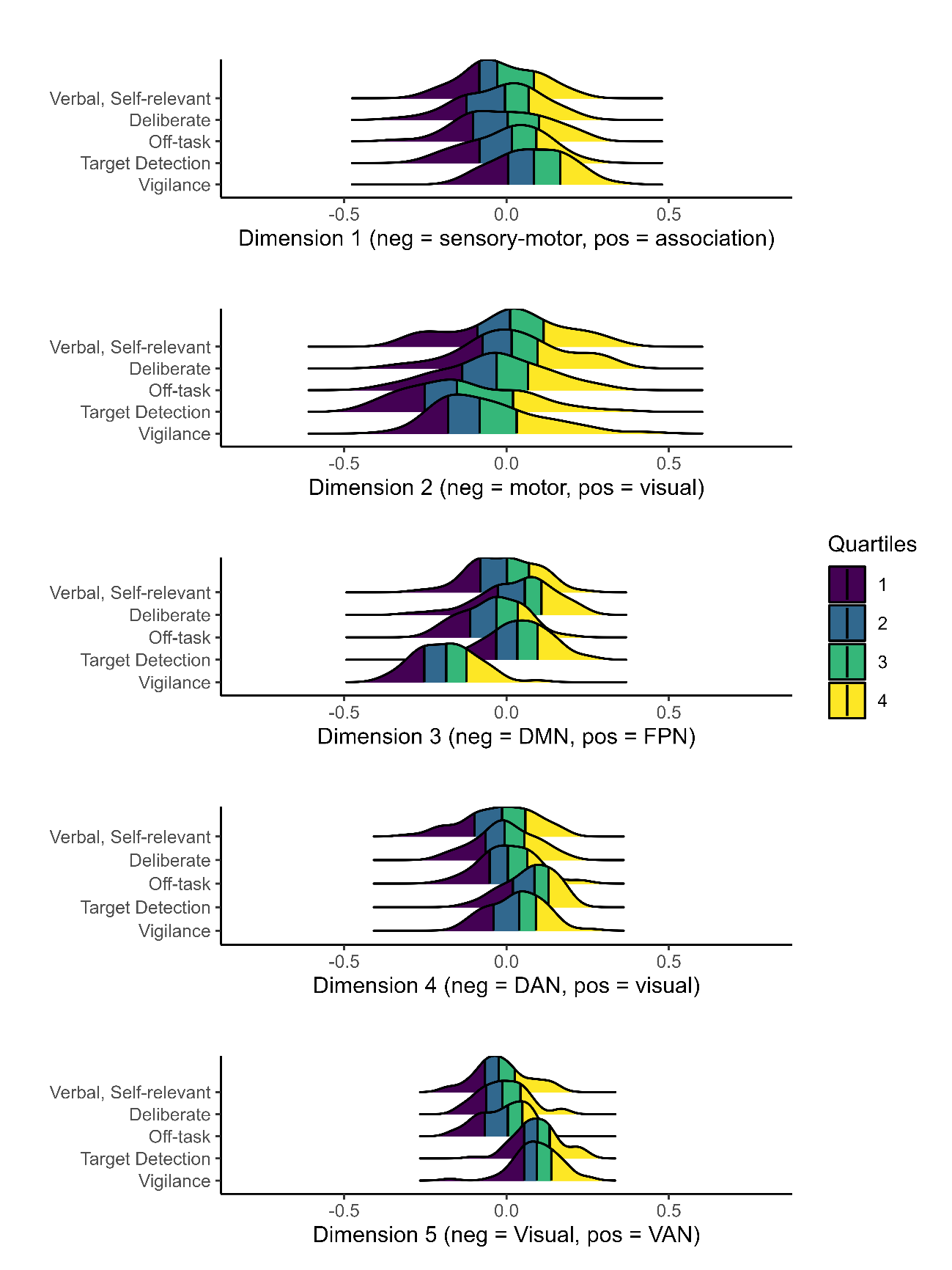


Supplementary Figure 1. Density ridge plots show the distribution of each task and experiential states’ coordinates along each dimension of the neural state space. The y-axis shows each of the five brain states examined in the current study (3 covert states, 2 overt states), and the x-axis shows the coordinate values along each dimension. The color indicates 25% Quartiles. N observations per brain state = 57. In this plot, ‘pos’ = positive end of dimension, ‘neg’ = negative end of dimension, DMN = default mode network, FPN = fronto-parietal network, DAN = dorsal attention network, VAN = ventral attention network.


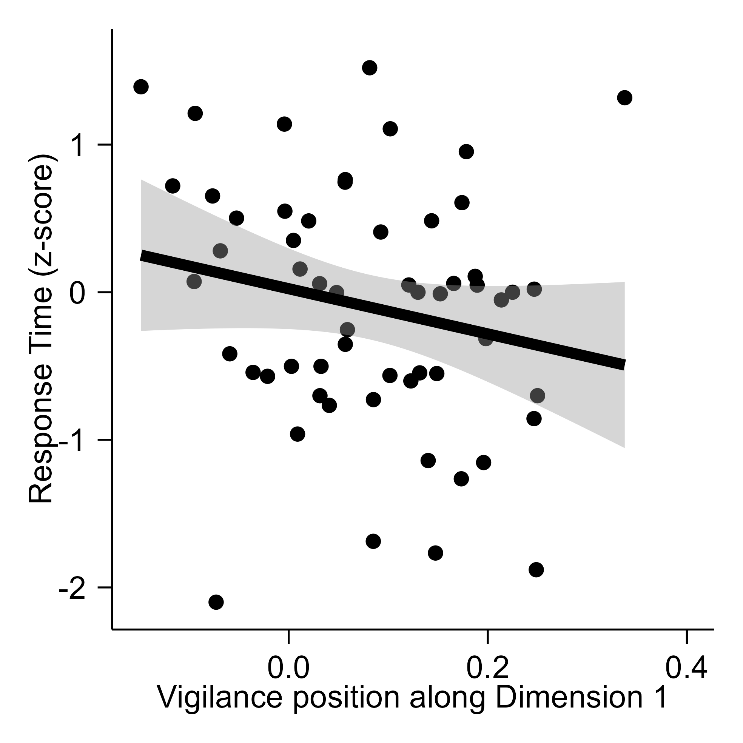


Supplementary Figure 2. Scatterplot shows the relationship between the position of vigilance states along dimension 1 of the neural state space and target detection response time. Individuals whose vigilance states fell towards the association end of dimension 1 tended to respond faster to targets. *Note:* although the F-stat for this effect was significant, the bootstrapped parameter estimate was not (see Supplementary Table 2).


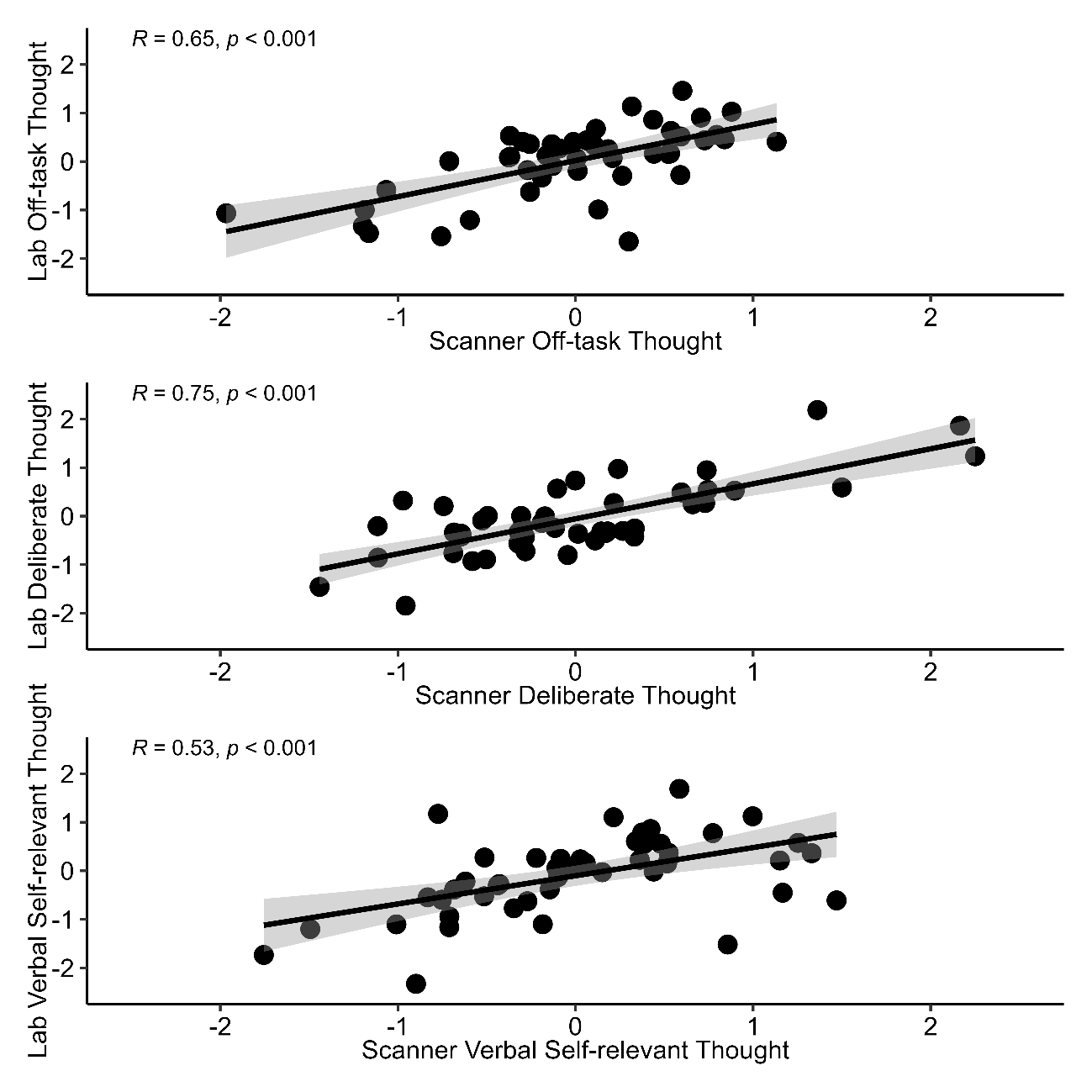


Supplementary Figure 3. Scatterplots show the correlations between average PCA scores derived from Principal Components Analysis (PCA) applied to the experience sampling data collected in 1) the lab (y-axis) and 2) the scanner (x-axis). Error bars represent 95% Confidence intervals. N observations = 46.

Supplementary Table 1. Bootstrapped estimates (n iterations = 1000) for linear mixed models comparing the location of overt task states (vigilance & target detection) along each dimension of the neural state space.

|  | **Dimension 1** | | | | **Dimension 2** | | | | **Dimension 3** | | | | **Dimension 4** | | | | **Dimension 5** | | | |
| --- | --- | --- | --- | --- | --- | --- | --- | --- | --- | --- | --- | --- | --- | --- | --- | --- | --- | --- | --- | --- |
| *Parameter* | *b* | *95 CI Low* | *95 CI High* | *p* | *b* | *95 CI Low* | *95 CI High* | *p* | *b* | *95 CI Low* | *95 CI High* | *p* | *b* | *95 CI Low* | *95 CI High* | *p* | *b* | *95 CI Low* | *95 CI High* | *p* |
| (Intercept) | -0.01 | -0.14 | 0.12 | .948 | -0.22 | -0.40 | -0.04 | .024 | -0.12 | -0.24 | -0.02 | .018 | 0.06 | -0.03 | 0.15 | .212 | 0.18 | 0.08 | 0.27 | <.001 |
| Vigilance | 0.04 | 0.02 | 0.05 | **<.001** | 0.03 | 0.01 | 0.06 | **<.001** | -0.11 | -0.12 | -0.09 | **<.001** | -0.02 | -0.03 | -0.01 | **<.001** | -0.00 | -0.01 | 0.01 | .772 |
| Female | 0.00 | -0.02 | 0.03 | .768 | 0.04 | 0.00 | 0.08 | .048 | -0.01 | -0.03 | 0.02 | .506 | 0.00 | -0.02 | 0.02 | .712 | -0.01 | -0.03 | 0.01 | .228 |
| Age | 0.00 | -0.00 | 0.01 | .430 | 0.00 | -0.01 | 0.01 | .478 | 0.00 | -0.00 | 0.01 | .536 | -0.00 | -0.00 | 0.00 | .934 | -0.00 | -0.01 | 0.00 | .148 |
| Movement | -0.01 | -0.08 | 0.07 | .904 | 0.13 | 0.03 | 0.24 | .018 | 0.03 | -0.03 | 0.10 | .324 | -0.03 | -0.08 | 0.03 | .356 | -0.03 | -0.09 | 0.02 | .218 |

*Note.* *P <* .05 highlighted in bold. Summed contrasts were used, meaning that the intercept reflects the grand mean of all conditions for each model, each factor level estimate reflects the difference between the factor level and the intercept.

Supplementary Table 2. Bootstrapped estimates (n iterations = 1000) for the multiple regression examining how the location of overt task states (vigilance and target detection) along each dimension of the neural state space relates to target detection reaction time.

| *Parameter* | *b* | *95 CI Low* | *95 CI High* | *p* |
| --- | --- | --- | --- | --- |
| (Intercept) | -0.17 | -1.91 | 1.19 | .800 |
| Vigilance along dimension 1 | -2.43 | -5.40 | 0.28 | .064 |
| Vigilance along dimension 2 | -0.77 | -3.12 | 1.44 | .444 |
| Vigilance along dimension 3 | -2.54 | -6.44 | 1.87 | .214 |
| Vigilance along dimension 4 | 1.67 | -2.56 | 6.29 | .416 |
| Vigilance along dimension 5 | 1.38 | -4.45 | 5.95 | .586 |
| Target detection along dimension 1 | 1.56 | -0.60 | 4.45 | .166 |
| Target detection along dimension 2 | 2.00 | 0.29 | 3.72 | **.016** |
| Target detection along dimension 3 | -1.18 | -3.64 | 1.79 | .332 |
| Target detection along dimension 4 | 3.09 | -1.59 | 7.19 | .168 |
| Target detection along dimension 5 | -4.34 | -9.16 | 0.48 | .078 |
| Age | 0.00 | -0.04 | 0.06 | .952 |
| Female | 0.12 | -0.14 | 0.41 | .338 |
| Mean Movement | 0.04 | -1.09 | 0.57 | .886 |

*Note*. *P <* .05 highlighted in bold. Reaction time was z-scored. Summed contrasts were used, meaning that the intercept reflects the grand mean of all conditions for each model, and each factor level estimate reflects the difference between the factor level and the intercept.

Supplementary Table 3. Bootstrapped estimates (n iterations = 1000) for linear mixed models comparing the location of covert experiential states (off-task episodic social cognition, deliberate task focus, and verbal self-relevant thought) along each dimension of the neural state space.

|  | **Dimension 1** | | | | **Dimension 2** | | | | **Dimension 3** | | | | **Dimension 4** | | | | **Dimension 5** | | | |
| --- | --- | --- | --- | --- | --- | --- | --- | --- | --- | --- | --- | --- | --- | --- | --- | --- | --- | --- | --- | --- |
| *Parameter* | *b* | *95 CI Low* | *95 CI High* | *p* | *b* | *95 CI Low* | *95 CI High* | *p* | *b* | *95 CI Low* | *95 CI High* | *p* | *b* | *95 CI Low* | *95 CI High* | *p* | *b* | *95 CI Low* | *95 CI High* | *p* |
| (Intercept) | 0.04 | -0.06 | 0.13 | .484 | -0.02 | -0.14 | 0.10 | .756 | -0.01 | -0.09 | 0.06 | .720 | -0.01 | -0.08 | 0.07 | .882 | 0.03 | -0.04 | 0.09 | .366 |
| Off-task | 0.01 | -0.01 | 0.03 | .414 | -0.04 | -0.07 | -0.00 | .032 | -0.04 | -0.06 | -0.02 | **<.001** | 0.01 | -0.01 | 0.03 | .200 | 0.00 | -0.01 | 0.02 | .810 |
| Deliberate | -0.00 | -0.03 | 0.02 | .734 | 0.02 | -0.01 | 0.06 | .174 | 0.04 | 0.02 | 0.06 | **<.001** | 0.00 | -0.02 | 0.02 | .826 | 0.00 | -0.01 | 0.02 | .760 |
| Female | -0.04 | -0.10 | 0.02 | .198 | 0.05 | -0.03 | 0.12 | .228 | -0.03 | -0.07 | 0.02 | .302 | -0.06 | -0.11 | -0.02 | .012 | -0.01 | -0.05 | 0.03 | .614 |
| Age | -0.00 | -0.02 | 0.02 | .956 | -0.01 | -0.04 | 0.02 | .504 | -0.01 | -0.02 | 0.01 | .452 | 0.00 | -0.01 | 0.02 | .514 | -0.00 | -0.02 | 0.01 | .692 |
| Movement | -0.00 | -0.01 | 0.00 | .518 | 0.00 | -0.00 | 0.01 | .982 | 0.00 | -0.00 | 0.00 | .564 | 0.00 | -0.00 | 0.00 | .520 | -0.00 | -0.00 | 0.00 | .242 |

*Note.* *P <* .05 highlighted in bold. Summed contrasts were used, meaning that the intercept reflects the grand mean of all conditions for each model, and each factor level estimate reflects the difference between the factor level and the intercept.

Supplementary Table 4. Bootstrapped estimates (n iterations = 1000) for the multiple regression examining how the location of covert experiential states (off-task social episodic cognition, deliberate task focus, and verbal self-relevant thought) along each dimension of the neural state space relate to target detection reaction time.

| *Parameter* | *b* | *95 CI Low* | *95 CI High* | *p* |  |
| --- | --- | --- | --- | --- | --- |
| (Intercept) | 0.27 | -2.58 | 2.90 | .796 |  |
| Off-task along dimension 1 | 1.05 | -1.61 | 3.80 | .382 |  |
| Off-task along dimension 2 | -2.25 | -7.13 | 2.63 | .358 |  |
| Off-task along dimension 3 | 1.59 | -3.23 | 6.51 | .476 |  |
| Off-task along dimension 4 | -0.40 | -6.09 | 7.24 | .908 |  |
| Off-task along dimension 5 | 1.24 | -2.35 | 4.43 | .432 |  |
| Deliberate along dimension 1 | 0.07 | -3.12 | 3.31 | .976 |  |
| Deliberate along dimension 2 | 0.89 | -2.07 | 4.14 | .562 |  |
| Deliberate along dimension 3 | 0.00 | -4.88 | 4.73 | 1.000 |  |
| Deliberate along dimension 4 | -2.33 | -6.65 | 2.12 | .298 |  |
| Deliberate along dimension 5 | -1.39 | -4.88 | 1.82 | .346 |  |
| Verbal Self along dimension 1 | -0.49 | -3.37 | 2.01 | .670 |  |
| Verbal Self along dimension 2 | 2.01 | -2.42 | 6.39 | .320 |  |
| Verbal Self along dimension 3 | -1.96 | -6.45 | 2.01 | .336 |  |
| Verbal Self along dimension 4 | 0.24 | -3.94 | 3.88 | .888 |  |
| Verbal Self along dimension 5 | -0.01 | -0.13 | 0.08 | .796 |  |
| Female | 0.20 | -0.15 | 0.59 | .272 |  |
| Mean Movement | -0.12 | -1.67 | 0.78 | .782 |  |

*Note*. Reaction time was z-scored. Summed contrasts were used, meaning that the intercept reflects the grand mean of all conditions for each model, and each factor level estimate reflects the difference between the factor level and the intercept.

Supplementary Table 5. Bootstrapped estimates (n iterations = 1000) for the multiple regression examining how experiential reports of covert states (off-task social episodic cognition, deliberate task focus, and verbal self-relevant thought) relate to target detection reaction time.

| *Parameter* | *b* | *Lower*  *95 CI* | *Upper*  *95 CI* | *p* |
| --- | --- | --- | --- | --- |
| (Intercept) | -0.36 | -1.60 | 0.75 | .556 |
| Off-task | 0.17 | -0.25 | 0.50 | .344 |
| Deliberate | -0.34 | -0.60 | -0.01 | **.046** |
| Verbal Self | 0.01 | -0.33 | 0.29 | .930 |
| Age | 0.01 | -0.03 | 0.06 | .696 |
| Female | 0.21 | -0.02 | 0.44 | .070 |
| Mean Movement | -0.09 | -1.09 | 0.39 | .664 |

*P* < .05 highlighted in bold. Reaction time was z-scored. Summed contrasts were used, meaning that the intercept reflects the grand mean of all conditions for each model, and each factor level estimate reflects the difference between the factor level and the intercept.

Supplementary Table 6. Estimated marginal means (EMM) of average activity level in each of the 7 Yeo Networks ^6^ for each overt task state (vigilance and target detection).

| State | Yeo Network | EMM | 95 CI Low | 95 CI High |
| --- | --- | --- | --- | --- |
| Vigilance | Visuospatial | -0.19 | -0.44 | 0.07 |
| Target | Visuospatial | -0.35 | -0.61 | -0.09 |
| Vigilance | Somatomotor | 0.12 | -0.14 | 0.38 |
| Target | Somatomotor | 0.18 | -0.08 | 0.43 |
| Vigilance | Dorsal Attention | -1.62 | -1.88 | -1.36 |
| Target | Dorsal Attention | -0.42 | -0.68 | -0.17 |
| Vigilance | Ventral Attention | 0.15 | -0.11 | 0.40 |
| Target | Ventral Attention | 1.03 | 0.77 | 1.29 |
| Vigilance | Limbic | 0.82 | 0.56 | 1.07 |
| Target | Limbic | 0.27 | 0.02 | 0.53 |
| Vigilance | Frontoparietal | -0.38 | -0.64 | -0.12 |
| Target | Frontoparietal | 0.32 | 0.06 | 0.58 |
| Vigilance | Default Mode | 1.08 | 0.82 | 1.33 |
| Target | Default Mode | 0.32 | 0.07 | 0.58 |

Supplementary Table 7. Estimated marginal means (EMM) of average activity level in each of the 7 Yeo Networks ^6^ for each covert experiential state (off-task social episodic cognition, deliberate task focus, and verbal self-relevant thought).

| State | Network | EMM | 95 CI High | 95 CI Low |
| --- | --- | --- | --- | --- |
| Off-task | Default Mode | 0.17 | 0.00 | 0.33 |
| Deliberate | Default Mode | -0.15 | -0.31 | 0.02 |
| Verbal Self | Default Mode | -0.06 | -0.22 | 0.10 |
| Off-task | Somatomotor | 0.17 | 0.01 | 0.34 |
| Deliberate | Somatomotor | -0.05 | -0.21 | 0.12 |
| Verbal Self | Somatomotor | 0.07 | -0.09 | 0.24 |
| Off-task | Limbic | 0.17 | 0.00 | 0.33 |
| Deliberate | Limbic | -0.09 | -0.25 | 0.08 |
| Verbal Self | Limbic | -0.01 | -0.17 | 0.15 |
| Off-task | Visuospatial | 0.19 | 0.03 | 0.36 |
| Deliberate | Visuospatial | -0.05 | -0.22 | 0.11 |
| Verbal Self | Visuospatial | 0.04 | -0.12 | 0.20 |
| Off-task | Dorsal Attention | 0.10 | -0.06 | 0.27 |
| Deliberate | Dorsal Attention | 0.04 | -0.13 | 0.20 |
| Verbal Self | Dorsal Attention | 0.09 | -0.08 | 0.25 |
| Off-task | Ventral Attention | 0.02 | -0.14 | 0.19 |
| Deliberate | Ventral Attention | 0.03 | -0.13 | 0.19 |
| Verbal Self | Ventral Attention | -0.03 | -0.19 | 0.14 |
| Off-task | Frontoparietal | 0.07 | -0.10 | 0.23 |
| Deliberate | Frontoparietal | -0.02 | -0.18 | 0.15 |
| Verbal Self | Frontoparietal | 0.00 | -0.16 | 0.16 |

Supplementary Table 8. Multidimensional Experience Sampling (mDES) items used in the current study to examine the contents and form of individuals’ ongoing thoughts during the task.

| Dimension | Statement | Scale low | Scale high |
| --- | --- | --- | --- |
| Task | My thoughts were focused on the task I was performing: | Not at all | Completely |
| Future | My thoughts involved future events: | Not at all | Completely |
| Past | My thoughts involved past events: | Not at all | Completely |
| Self | My thoughts involved myself: | Not at all | Completely |
| Person | My thoughts involved other people: | Not at all | Completely |
| Emotion | The emotion of my thoughts was: | Negative | Positive |
| Modality | My thoughts were in the form of: | Images | Words |
| Detail | My thoughts were detailed and specific: | Not at all | Completely |
| Deliberate | My thoughts were: | Spontaneous | Deliberate |
| Problem | I was thinking about solutions to problems (or goals): | Not at all | Completely |
| Diverse | My thoughts were: | One topic | Many topics |
| Intrusive | My thoughts were intrusive: | Not at all | Completely |
| Source | My thoughts were linked to information from: | Environment | Memory |

References

1 R: A language and environment for statistical computing. v. 4.1.1. (R Foundation for Statistical Computing, Vienna, Austria, 2021).

2 Bates, D., Maechler, M., Bolker, B. & Walker, S. Fitting Linear Mixed-Effects Models Using lme4. *Journal of Statistical Software* **67**, 1-48 (2015).

3 Kuznetsova, A., Brockhoff, P. B. & Christensen, R. H. lmerTest Package: Tests in Linear Mixed Effects Models. *Journal of statistical software* **82**, 1-26 (2017).

4 Singmann, H. & Kellen, D. An introduction to mixed models for experimental psychology. *New methods in cognitive psychology* **28**, 4-31 (2019).

5 emmeans: Estimated Marginal Means, aka Least-Squares Means. v. 1.7.0. (2021).

6 Yeo, B. T. *et al.* The organization of the human cerebral cortex estimated by intrinsic functional connectivity. *Journal of neurophysiology* (2011).
